# Characterisation of bacterial growth and antimicrobial susceptibility patterns in canine urinary tract infections

**DOI:** 10.1186/s12917-014-0217-4

**Published:** 2014-09-24

**Authors:** Ulrika Windahl, Bodil Ström Holst, Ann Nyman, Ulrika Grönlund, Björn Bengtsson

**Affiliations:** Department of Animal Health and Antimicrobial Strategies, National Veterinary Institute, SVA SE-751 89 Uppsala, Sweden; Department of Clinical Sciences, Swedish University of Agricultural Sciences, SE-75007 Uppsala, Sweden

**Keywords:** Urinary tract, Bacterial infection, Dog, Antimicrobial resistance, Extended spectrum cephalosporins

## Abstract

**Background:**

Bacterial urinary tract infection (UTI) is a common reason for antimicrobial therapy in dogs.

A reported increase in multi-drug resistance in canine bacterial pathogens, including resistance to extended-spectrum cephalosporins (ESC) is of concern as antimicrobial resistance complicates therapy in dogs. In addition, it is a possible public health concern.

The objectives of this study were to investigate the relative prevalence of pathogens in urine samples from dogs with urinary tract infection sampled at referral hospitals, clinics and mixed veterinary practices and to investigate if this was influenced by sample material or by contamination of the culture. The second objective was to assess the susceptibility patterns to clinically relevant antimicrobials and to investigate if this was influenced by whether the samples originated from smaller clinics or from referral hospitals and to perform active screening for the presence of Enterobacteriaceae resistant to ESC.

**Results:**

*Escherichia coli* was the most frequently isolated pathogen (68%) followed by staphylococci (11%).

*E. coli* isolates were found significantly more often in pure culture than in contaminated samples. *Staphylococcus pseudintermedius* and *Staphylococcus aureus* isolates were significantly more prevalent in pre-incubated samples compared to samples submitted as non-incubated media.

Susceptibility to the majority of the tested first-line antimicrobials was common. Multiresistance was rare, and these isolates were all susceptible to at least one relevant antimicrobial. Isolates in samples from small animal clinics or mixed veterinary practices were less likely to be susceptible compared to isolates originating from referral animal hospitals. ESC-resistant Enterobacteriacae isolates were found in one per cent of the positive cultures. Bacteria with transferable ESC resistance were confirmed in one dog. The gene demonstrated was *bla*_CMY2_.

**Conclusions:**

Choice of sample material might influence the possibility of detecting *Staphylococcus pseudintermedius* and *Staphylococcus aureus* isolates in clinical cases of UTI in dogs. Based on the study results, use of first-line antimicrobials is a rational empirical antimicrobial therapy for the studied dog population.

*E. coli* was the most prevalent pathogen, but prevalence of infection with ESC resistant Enterobacteriaceae including *E. coli* was low, as such isolates were found in only one per cent of the positive cultures.

## Background

Bacterial urinary tract infection (UTI) is a common clinical problem in dogs and among the most common reason for antimicrobial therapy, with approximately 14% of all dogs having at least one episode of UTI during their lifetime [1]. An increase in multi-drug resistance in canine bacterial pathogens, including resistance to extended-spectrum cephalosporins (ESC) associated with the production of extended-spectrum beta-lactamases (ESBL) or AmpC beta-lactamases, in urinary isolates has been reported [2-6]. Increasing antimicrobial resistance in canine bacterial pathogens is of concern as it complicates therapy in dogs. In addition, it is a public health concern when the pathogens are zoonotic, or the location of the resistance genes enables transfer between bacteria of animal and human origin [5,7].

Prudent use of antimicrobials is an important step in reducing the emergence of antimicrobial resistance. Microbiological culture combined with susceptibility testing is the cornerstone of UTI diagnosis and the best instrument for guiding treatment decisions in individual dogs [8,9]. However, empirical treatment is often necessary before the culture and susceptibility results are available. In the context of canine UTIs, prudent use thus includes considering likely pathogens and their susceptibility patterns when choosing empirical treatment [8].

The first objective of this study was to estimate the relative prevalence of uropathogens in dogs with urinary tract infections and to investigate if this was influenced by sample material or by contamination of the culture. The second objective was to assess the susceptibility patterns to clinically relevant antimicrobials and to investigate if this was influenced by whether the samples originated from smaller veterinary clinics or from referral hospitals and to perform active screening for the presence of Enterobacteriaceae resistant to ESC. The results were intended to aid in assessing culture results from urinary tract infections in dogs in clinical practice and to provide a basis for rational empirical antimicrobial therapy. Moreover, the results were to provide a basis for future monitoring of trends in antimicrobial resistance.

## Methods

### Samples

The urine samples were routine samples collected from dogs with clinical signs of urinary tract infection and submitted by the attending veterinarian to SVA (National Veterinary Institute, Sweden) for culture and susceptibility testing during a ten-month study period (March-December 2009).

### Culture

The samples were processed on the day of arrival to SVA. The referred material (urine sent in a sterile container, bacterial swabs, representative colonies on dipslide, or agar plate bacterial cultures) were inoculated and spread onto horse blood agar (National Veterinary Institute, Uppsala, Sweden) and onto Cysteine Lactose Electrolyte Deficient agar (Oxoid Ltd, Merck KGaA). All inoculated agar plates were incubated at 37°C for 24 to 48 h until adequate growth was present. Bacterial identification was based on colony type and morphology, gram staining characteristics and standard biochemical tests [10-12]. In addition, material from each submitted sample (0.1 mL of urine or, when the submitted sample consisted of pre-incubated cultures, the colony material collected by streaking across the agar using a calibrated loop), was spread onto MacConkey agar (National Veterinary Institute, Uppsala, Sweden) supplemented with cefotaxime (1 mg/L) (Sigma-Aldrich) with the objective to screen for Enterobacteriaceae resistant to ESC.

After incubation at 37°C for 24 to 48 h until adequate growth, colonies that based on type and morphology were identified as possible *Escherichia coli*, *Klebsiella* spp. or *Enterobacter* spp. were selected and spread on horse-blood agar (5% v/v) and incubated overnight. To identify relevant isolates when growth on the selective media was rich, colonies were spread onto horse-blood agar with a cefotaxime disc (30 μg, CTO166B Oxoid Ltd) on the agar surface. The final identification of up to eight representative isolates per agar plate was based on production of tryptophanase (indole) and ß-glucuronidase (p-nitrophenyl- ß-D- glucopyranosiduronic acid, PGUA) and the analytical profile index API 20 E® identification system (bioMérieux).

### Antimicrobial susceptibility

Antimicrobial susceptibility was tested by determination of minimum inhibitory concentration (MIC) using broth microdilution following the standards of the Clinical and Laboratory Standards Institute [13] and using VetMIC panels (SVA, Uppsala, Sweden). *Escherichia coli* ATCC 25922 and *Enterococcus faecalis* ATCC 29212, *Staphylococcus aureus* ATCC 29213 and *Pseudomonas aeruginosa* ATCC 27853 were used for quality control. In addition, staphylococci were tested for beta-lactamase production by the “clover-leaf” method as described by Bryan and Godfrey [14]. Isolates of presumptive uropathogens were tested for susceptibility to antimicrobials relevant for the respective bacterial species [15]. Specifically, *E. coli*, *Klebsiella* spp. and *P. mirabilis* were tested for susceptibility to cefotaxime as an indicator of ESC-resistance, and *S. pseudintermedius* and *S. aureus* were tested for susceptibility to oxacillin as an indicator of methicillin resistance. Isolates were classified as susceptible to an antimicrobial according to MIC breakpoints for bacteria from animals issued by CLSI [16]. CLSI breakpoints for bacteria from humans were used for nitrofurantoin and cefotaxime as there are no interpretive criteria for bacteria from animals for these antimicrobials [17]. Only isolates of staphylococci not producing beta-lactamase were classified as susceptible to ampicillin and penicillin.

An isolate was classified as multiresistant (MDR) if it was intermediately susceptible or resistant to three or more antimicrobial classes according to CLSI breakpoints. For the classification of MDR in *E. coli*, *Klebsiella* spp. and *P. mirabilis,* ampicillin and amoxicillin-clavulanic acid were considered one antimicrobial class. For the classification of staphylococci, streptococci and enterococci, ampicillin, amoxicillin-clavulanic acid, cephalothin and penicillin were considered one antimicrobial class. Antimicrobials to which a bacterial species has inherently low susceptibility were not included in the classification of MDR. This applies to ampicillin and nitrofurantoin in *Klebsiella* spp., nitrofurantoin and tetracycline in *P. mirabilis* and to enrofloxacin and gentamicin in streptococci. Enterococci have constitutively low susceptibility to several of the antimicrobials studied, and only ampicillin, tetracycline, trimethoprim-sulphametoxazole and nitrofurantoin were considered in the evaluation of MDR in enterococci.

Isolates of *E. coli, Klebsiella* spp. and *Enterobacter* spp*.* recovered from the screening for ESC-resistance were tested for susceptibility to cefotaxime by broth microdilution as described above. Isolates with cefotaxime MIC >1 mg/L were further analysed for the production of ESBL or AmpC by the double disc diffusion test according to CLSI [13].

### Genotyping

Presumptive methicillin resistant staphylococci, based on oxacillin MIC, were tested for presence of the *mec*A gene by PCR as described by Strommenger et al. [18]. To differentiate isolates of Enterobacteriaceae with chromosomal AmpC production from isolates carrying plasmidic AmpC, a multiplex PCR assay for the plasmid carrying the beta-lactamase families MOX, CIT, DHA, ACC, EBC and FOX was used [19]. In isolates shown to carry plasmidic AmpC, the gene variants were determined by sequencing using in-house primers (bla_cmy-2_ 5′- AAATCGTTATGCTSCGCTCT and 5′- CATGGGATTTTCCTTGCTGT) and Big-Dye™ v1.1.

### Statistical analyses

The statistical analyses were performed using Stata Software (StataCorp. 2012; Stata Statistical Software: Release 11.2; College Station, TX, USA: StataCorpLP). Descriptive statistics were used to describe the uropathogens isolated in pure and contaminated culture, in different sample media (pre-incubated cultures or non-incubated samples), and from different submission categories. Univariable associations between findings of the isolated uropathogens and the three explanatory variables; submission category, sample media (whether the samples were sent in as pre-incubated cultures or not), and if the samples were in pure or contaminated cultures were investigated using Fisher’s exact-test, the χ^2^-test and univariable logistic regression analysis. The same tests were used for investigation of aassociations between submission category and if samples were in pure or contaminated culture, as well as for associations between submission category and the use of different sample media. The same univariable tests were used to investigate if antimicrobial susceptibility in isolates varied by submission category. Moreover, for every uropathogen a multivariable logistic regression analysis was performed using a manual stepwise backward variable selection procedure in where the initial models included all three explanatory variables as main effects. Collinearity between the explanatory variables was assessed pair-wise by Spearman rank correlations. If there was proof of collinearity (r ≥ 0.70) the variable with lowest *P*-value in the univariable analysis was selected. All plausible two-way interactions between the significant main effects were tested. Variables with a significant association (*P* < 0.05) with the dependent variable were kept in the model. The model fit was evaluated with the Hosmer-Lemeshow goodness-of-fit test and by visual examination of diagnostic plots as outlined by Hosmer and Lemeshow [20]. For all the analyses performed, the level of statistical significance was set at *P* ≤0.05.

### Ethics

All samples were collected during routine diagnostics. The study was approved by the Swedish Board of Agriculture and the Ethical Committee on Animal Experiments in Uppsala.

## Results

### Population characteristics

Of the in total 1042 submitted samples, 469 (45%) originated from four small animal referral hospitals that submitted 94–129 samples each. Approximately a quarter of the samples (n = 277; 27%) were referred by ten separate small animal clinics, each contributing 11–55 samples. The remaining 296 samples (28%) were from 115 mixed veterinary practices, of which none submitted more than ten urinary samples. These three submission categories; small animal referral hospitals, small animal clinics and mixed veterinary practices were labelled A, B and C, respectively (Table 1).

**Table 1:** **Findings of uropathogens in submitted urinary samples presented by submission category, sample material and growth**

	**Submission category**			**Sample material**		**Growth**	
	**A**	**B**	**C**	**Pre-incubated**	**Non-incubated**	**Pure**	**Conta-minated**
	**% (n)**	**% (n)**	**% (n)**	**% (n)**	**% (n)**	**% (n)**	**% (n)**
**Positive cultures individual dogs (n = 623)**	45 (284)	25 (154)	30 (185)	68 (423)	32 (200)	60 (375)	40 (248)
**Positive cultures repeated samples (n = 48)**	52 (25)	33 (16)	13 (7)	83 (40)	17 (8)	58 (28)	42 (20)
**Samples without significant growth (n = 371)**	43 (160)	29 (107)	28 (104)	26 (95)	74 (276)	0	0

The relative prevalence (%) is presented with the number of samples shown inside brackets.

### Characterisation of bacterial growth

Of the submitted samples, 961 were found to be from individual dogs. A specific urinary pathogen was isolated in 623 (65%) of these. Culture of 338 samples (35%) yielded either no bacterial growth or only insignificant non-specific growth of contaminants, including 20 samples where more than one possible pathogen was found together with growth of contaminants that prohibited further confirmation of the suspected findings (Table 1). Eighty-one of the submitted samples were found to be repeated samples from dogs already included in the study. Of these, 48 positive cultures were found to be repeated samples from 40 dogs, and 33 samples yielded no growth or only non-specific growth. The 48 repeated samples with positive cultures were evaluated separately.

Significantly more pre-incubated (68%, n = 423) than non-incubated samples (32%, n = 200) yielded positive culture results (*P* < 0-001). Samples that were incubated prior to referral were six times more likely to yield positive growth.

### Positive cultures from individual dogs

Seven different urinary pathogens were identified in the 623 positive cultures with a specific urinary pathogen. *Escherichia coli* was the most prevalent, being identified in 429 (68.9%) of the samples, followed by *S. pseudintermedius* (9.6%, n = 60), *P. mirabilis* (8.8%, n = 55), beta haemolytic *Streptococcus* spp. (5.6%, n =35), *Enterococcus* spp. (3.7%, n =23), *Klebsiella* spp. (1.8%, n = 11) and *S. aureus* (1.6%, n =10) (Table 2).

**Table 2:** **Relative prevalence of uropathogens in non-repeated urinary samples from individual dogs**

**Submission category A referral hospitals 45%, (n = 284)**	***E.coli***	***S. pseudo-intermedius***	***Proteus mirabilis***	**β-haemolytic** ***Streptococcus*** **spp.**	***S. aureus***	***Enterococcus*** **spp.**	***Klebsiella*** **spp.**
**Pre-incubated 87%, (n = 248)**							
Pure growth, (n = 174)	72 (131)	57 (16)	77 (10)	78 (7)	57 (4)	71 (5)	33 (1)
Contaminated growth, (n = 74)	28 (50)	43 (12)	23 (3)	22 (2)	43 (3)	29 (2)	67 (2)
**Non-incubated, 13% (n = 36)**							
Pure growth, (n = 22)	74 (17)	50 (2)	25 (1)	50 (2)	-	0 (0)	-
Contaminated growth, (n = 14)	26 (6)	50 (2)	75 (3)	50 (2)	-	100 (1)	-
**Submission category B small animal clinics ** **25%, (n = 154)**	***E.coli***	***S. pseudo-intermedius***	***Proteus mirabilis***	**β-haemolytic** ***Streptococcus*** **spp.**	***S. aureus***	***Enterococcus*** **spp.**	***Klebsiella*** **spp.**
**Pre-incubated, 56%, (n = 87)**							
Pure growth, (n = 53)	68 (42)	67 (6)	33 (3)	0 (0)	0 (0)	100 (2)	0 (0)
Contaminated growth, (n = 34)	32 (20)	33 (3)	67 (6)	100 (2)	100 (1)	0 (0)	100 (2)
**Non-incubated, 44%, (n = 67)**							
Pure growth, (n = 34)	52 (25)	67 (2)	75 (6)	0 (0)	-	33 (1)	-
Contaminated growth, (n = 33)	48 (23)	33 (1)	25 (2)	100 (5)	-	67 (2)	-
**Submission category C mixed veterinary practices ** **30%, (n =185)**	***E.coli***	***S. pseudo-intermedius***	***Proteus mirabilis***	**β-haemolytic** ***Streptococcus*** **spp.**	***S. aureus***	***Enterococcus*** **spp.**	***Klebsiella*** **spp.**
**Pre-incubated, 48%, (n = 88)**							
Pure growth, (n = 45)	52 (29)	50 (6)	67 (6)	25 (1)	100 (2)	33 (1)	0 (0)
Contaminated growth, (n = 43)	48 (27)	50 (6)	33 (3)	75 (3)	0 (0)	67 (2)	100 (2)
**Non-incubated, 52%, (n = 97)**							
Pure growth, (n = 47)	51 (30)	50 (2)	50 (6)	36 (4)	-	43 (3)	50 (2)
Contaminated growth, (n = 50)	49 (29)	50 (2)	50 (6)	64 (7)	-	57 (4)	50 (2)
**Total**	68 (429)	10 (60)	9 (55)	5 (35)	2 (10)	4 (23)	2 (11)

The relative prevalence (%) is presented by the three submission categories; small animal referral hospitals, small animal clinics and mixed veterinary practices are labelled A, B and C, respectively, as well as by submitted sample material and by pure- or contaminated growth. The number of samples is shown inside brackets.

Forty-six per cent (n = 284) of the 623 cultures with a specific urinary pathogen originated from submission category A, 25% (n = 154) from category B and 30% (n =185) from category C. Approximately 68% (n = 423) of the samples were submitted to the laboratory as pre-incubated cultures on dipslides (42%) or agar plates (26%). The 200 non-incubated samples were submitted in the form of urine collected in a sterile container (19%) or as bacterial swabs dipped in urine (13%). Submission category A sent in more samples using pre-incubated sample media than submission groups B and C (*P* < 0.001 and *P* < 0.001, respectively).

Of the 623 samples, 60% (n = 375) were in pure growth, and 40% (n = 248) were in contaminated growth (Table 1). Significantly more samples from submission category A were in pure culture compared to submission categories B (*P* < 0.01) and C (*P* < 0.001). Significantly more pre-incubated than non-incubated samples were in pure culture (P < 0.01) compared to contaminated culture. No significant differences were observed between the pre-incubated or non-incubated samples and the pure or contaminated samples when submission category was considered (Table 2).

The results of the multivariable logistic regression analyses of associations between the bacterial findings and the explanatory variables (submission category, submitted sample media and whether the samples were contaminated) differed between the uropathogens. The only multivariable model where more than one explanatory variable was significantly associated with the analysed uropathogen was the model of association with findings of beta haemolytic *Streptococcus* spp. Hence, the results for all other uropathogens are from the univariable logistic regression analyses.

There was a higher probability of finding *S. pseudintermedius* in pre-incubated sample media (n = 49) compared to non-incubated sample media (OR = 2.2; P = 0.019; n = 20). The use of pre-incubated sample media was associated with a lower probability of finding beta haemolytic *Streptococcus* spp. (OR = 0.4; *P* = 0.004; n = 15) compared to non-incubated sample media (n = 20). The ten *S. aureus* isolates were all found in pre-incubated samples. The probability of finding *E. coli* was lower (OR = 0.6; *P* = 0.005) in contaminated cultures (n = 155) than in pure cultures (n = 274), while the probability of finding beta haemolytic *Streptococcus* spp. (OR = 2.1; *P* = 0.04; n = 21) or *Klebsiella* spp. (OR = 4.1; *P* = 0.04 n = 8) was higher in contaminated cultures compared to pure cultures (n = 14 and n = 3, respectively).

### Positive cultures from repeated samples

The same pathogen was isolated on both occasions in all 48 repeated sample occasions with positive culture results. The bacteria isolated were *E. coli* (81.2%, n = 39), *S. pseudintermedius* (6.3%, n = 3)*, P. mirabilis* (6.3%, n = 3)%, *Klebsiella* spp. (4.2%, n = 2)%), and *Enterococcus* spp. (2.0%, n = 1).

All but four samples were submitted by the same veterinary practice, clinic or hospital that sampled the dog the first time. Approximately half of the samples (52%, n = 25) originated from category A, one third (33%, n = 16) were from category B and 14% (n = 7) were from category C. For all but two dogs, the submitted sample material did not differ between the sample occasions, and 83% (n = 40) of the samples were submitted as pre-incubated cultures.

### Antimicrobial susceptibility patterns in positive cultures from individual dogs

Of the gram-negative pathogens, *E. coli* had the overall highest percentage of susceptibility (Table 3)*.* Most (79%) *E. coli* isolates were susceptible to all the tested antimicrobials. Susceptibility to the individual antimicrobials varied between 87 and approximately 100%, with reduced susceptibility to aminopenicillins (ampicillin and amoxicillin-clavulanic acid) being the most common trait (Table 3). Seventeen *E. coli* isolates (4%) were multiresistant (Table 4). Eleven of these isolates were susceptible both to first generation fluoroquinolones (enrofloxacin) and nitrofurantoin. The remaining six multiresistant isolates were susceptible to at least one antimicrobial, which was usually nitrofurantoin.

**Table 3:** **Antimicrobial susceptibility (%) of** ***E. coli*** **,** ***Klebsiella*** **spp. and** ***P. mirabilis*** **in non-repeated samples**

**Antimicrobial**	**Breakpoint S**	***Escherichia coli***				***Klebsiella*** **spp.**				***Proteus mirabilis***			
		**All**	**A**	**B**	**C**	**All**	**A**	**B**	**C**	**All**	**A**	**B**	**C**
	**(mg/L)**	**(429)**	**(204)**	**(110)**	**(115)**	**(11)**	**(3)**	**(2)**	**(69**	**(55)**	**(17)**	**(17)**	**(21)**
Ampicillin	≤ 8	87.9	91.2	85.5	84.3	0.0	0.0	0.0	0.0	90.9	88.2	94.1	90.5
Amoxicillin/Clavulanic acid	≤ 8	87.2	89.7	85.5	84.3	72.7	66.7	100	66.7	94.5	100	94.1	90.5
Cefotaxime	≤ 1	99.8	99.5	100	100	100	100	100	100	98.2	100	94.1	100
Gentamicin	≤ 2	94.9	95.1	93.6	95.7	90.9	100	100	83.3	83.6	82.4	82.4	85.7
Enrofloxacin	≤ 0.5	97.9	98.5	95.5	99.1	90.9	100	100	83.3	96.4	100	88.2	100
Tetracycline	≤ 4	92.1	95.1	90.0	88.7	100	100	100	100	5.5	5.9	5.9	4.8
Trimethoprim/Sulphametoxazole	≤ 2/38	92.3	96.6	89.1	87.8	90.9	100	100	83.3	89.1	88.2	88.2	90.5
Nitrofurantoin	≤ 32	98.4	99.5	98.2	96.5	27.3	33.3	50.0	16.7	9.1	5.9	11.8	9.5

Breakpoints for susceptibility (BP S) (mg/L) are indicated. The number of samples is shown inside brackets. The three submission categories; small animal referral hospitals, small animal clinics and mixed veterinary practices are labelled A, B and C, respectively.

**Table 4:** **Resistance phenotypes of the 17 multidrugresistant** ***E. coli*** **isolates**

**Number of isolates**	**Ampicillin**	**Gentamicin**	**Enrofloxacin**	**Tetracycline**	**Trimethoprim/Sulphametoxazole**	**Nitrofurantoin**
3	**R**	**R**	**R**	**R**	**R**	
1	**R**	**R**	**R**	**R**		
1	**R**			**R**	**R**	**R**
11	**R**			**R**	**R**	
1	**R**		**R**	**R**		

The letter “R” in bold indicates resistance.

The majority (73 to 95%) of *Klebsiella* spp. and *P. mirabilis* isolates were susceptible to amoxicillin-clavulanic acid, gentamicin, enrofloxacin and trimethoprim-sulfamethoxazole. Ninety-one per cent of *P. mirabilis* isolates were susceptible to ampicillin. Constitutive resistance was reflected in the low number of isolates being susceptible to nitrofurantoin in both species (27% and 9%, respectively), in susceptibility to tetracycline being observed in less than 6% of the *P. mirabilis* isolates, and by none of the *Klebsiella* spp. isolates being susceptible to ampicillin (Table 3). No *Klebsiella* spp. isolates were susceptible to ampicillin. Disregarding constitutive resistance, no *Klebsiella* spp. isolates were multiresistant, although one isolate was neither susceptible to gentamicin nor to trimethoprim-sulphametoxazole. Only one *P. mirabilis* isolate (2%) was multiresistant when constitutive resistance to tetracycline and nitrofurantoin was disregarded. The isolate was not susceptible to ampicillin, gentamicin or trimethoprim-sulphametoxazole. A reduced susceptibility to cefotaxime, MIC >1 mg/L was detected in one isolate each of *E. coli* and *P. mirabilis*. Both of these isolates produced AmpC beta-lactamase. Further testing for the genotype was not performed.

The screening for isolates with ESC-resistance by culture on cefotaxime supplemented media yielded growth of *E. coli* (three samples), *Enterobacter cloacae* and *Enterobacter aerogenes* (two samples each). All seven isolates had cefotaxime MIC ≥ 2 mg/L and were confirmed as AmpC producers by phenotypic tests. A plasmidic AmpC gene was confirmed in only one isolate, which was an *E. coli* isolate carrying a gene of the CIT group that was confirmed as *bla*_cmy-2_ by sequencing. This isolate was obtained from the same sample as the *E. coli* isolate described above with reduced susceptibility to cefotaxime found on non-selective culture.

Only 10% of *S. pseudintermedius* and *S. aureus* isolates were susceptible to penicillin and ampicillin; 90% were resistant by production of beta-lactamase. Susceptibility to other antimicrobials ranged from 87 to 100%, with the exception of tetracycline, to which about 65% of the isolates were susceptible (Table 5). One *S. pseudintermedius* isolate had oxacillin MIC >2 mg/L and was confirmed as a *mec*A carrier. None of the *S. aureus* isolates but six isolates of *S. pseudintermedius* (10%) were multiresistant, including the methicillin resistant isolate (Table 6). Beta-haemolytic streptococci were uniformly susceptible to penicillin, amoxicillin-clavulanic acid, trimethoprim-sulphametoxazole and nitrofurantoin. The proportion of isolates susceptible to tetracycline or enrofloxacin was lower, 64% and 15%, respectively (Table 5). No isolate was multiresistant. Enterococci were the least susceptible of the isolated pathogens if all tested antimicrobials are considered (Table 5). However, susceptibility to ampicillin, amoxicillin-clavulanic acid, trimethoprim**-**sulphametoxazole and nitrofurantoin was high, with more than 80% of all the isolates being susceptible. Two isolates (9%) were resistant to three or more of these antimicrobials (Table 5).

**Table 5:** **Antimicrobial susceptibility (%) of** ***S. pseudintermedius*** **,** ***S. aureus*** **, beta haemolytic streptococci and enterococci in non-repeated samples**

**Antimicrobial**	***Staphylococcus pseudintermedius***		***Staphylococcus aureus***		**Beta-hemolytic streptococci**		**Enterococci**	
	**(n = 60)**		**(n = 10)**		**(n = 33)**		**(n = 55)**	
	**BP S**	**% S**	**BP S**	**% S**	**BP S**	**% S**	**BP S**	**% S**
Penicillin	β-lact^a^	10.0	β-lact	30.0	≤ 0.12	100	≤ 8	NR
Ampicillin	β-lact	10.0	β-lact	30.0	≤ 0.25	NR	≤ 8	87.0
Amoxicillin/Clavulanic acid	≤ 8	96.7	≤ 8	100	≤ 8	100	≤ 8	82.6
Cephalothin	≤ 2	98.3	≤ 2	100	≤ 2	100	≤ 2	13.0
Oxacillin	≤ 0.25	NR^b^	≤ 2	100	-	-	-	-
Gentamicin	≤ 2	98.3	≤ 2	100	≤ 2	0.0	≤ 2	30.4
Erythromycin	≤ 0.5	86.7	≤ 0.5	100	≤ 0.25	NR	≤ 0.5	34.8
Enrofloxacin	≤ 0.5	98.3	≤ 0.5	100	≤ 0.5	15.2	≤ 0.5	47.8
Tetracycline	≤ 4	66.7	≤ 4	90.0	≤ 2	63.6	≤ 4	65.2
Trimethoprim/Sulphametoxazole	≤ 2/38	95.0	≤ 2/38	100	≤ 2/38	100	≤ 2/38	87.0
Nitrofurantoin	≤ 32	100	≤ 32	100	≤ 32	100	≤ 32	82.6

Breakpoints for susceptibility (BP S) (mg/L) are indicated. The number of samples is shown inside brackets. Results from the susceptibility testing of one beta-haemolytic streptococcus isolate were lost and are not presented.

^a^β-lact = beta-lactamase production.

^b^NR = not relevant since the BP is outside of the range of concentrations tested.

**Table 6:** **Resistance phenotypes of the six multidrugresistant** ***S. pseudintermedius*** **isolates**

**Number of isolates**	**Penicillin and Aminopenicillins**	**Cephalotin**	**Genta-micin**	**Erythro- mycin**	**Enro-floxacin**	**Tetra-cycline**	**Trimethoprim/ Sulphametoxazole**
2	**R**		S	**R**	S	**R**	S
1	**R**		S	S	S	**R**	**R**
1	**R**		S	**R**	S	**R**	S
1	**R**		S	**R**	S	**R**	**R**
1^a^	**R**	**R**	**R**	**R**	**R**	S	**R**

The letter “R” in bold indicates resistance.

^a^ *mecA* carrier.

Comparison of susceptibility patterns between the three submission categories showed that the probability of finding trimethoprim-sulphametoxazole resistant *E. coli* was higher for submission categories B and C compared to submission group A (OR = 3.4; *P* = 0.01 and OR = 3.9; *P* = 0.004, respectively). The probability of finding tetracycline resistant *E. coli* was higher (OR = 2.5; *P* = 0.04) for submission category C than for submission category A. *Escherichia coli* from submission category C were more likely to be multiresistant than isolates from category A (OR = 2.7; *P* = 0.02). There was a trend for isolates from category B to be multiresistant to a greater extent than isolates from category A, but the difference was not significant (OR = 2.4; P = 0.052). There were no associations between submission category and antimicrobial susceptibility of *P. mirabilis* or beta-haemolytic streptococci, and the small number of resistant isolates precluded a statistical assessment of such associations for *S. pseudintermedius*, *S. aureus, Klebsiella* spp. or *Enterococcus spp*.

### Antimicrobial susceptibility patterns in positive cultures from repeated samples

Eighty-three per cent (n = 32) of the 39 *E. coli* isolates in cultures of the repeated samples were susceptible to all the tested antimicrobials. In six of the seven cultures yielding *E. coli* isolates with resistance traits, the isolates were less susceptible than on the first sample occasion. Four isolates were multiresistant (10% of the 39 *E. coli* isolates). Multiresistance was already present in the first isolates from the three dogs involved, but the isolates from the repeated samples had additional resistance to trimethoprim-sulphametoxazole. No Enterobacteriaceae resistant to ESC were isolated.

In eight of the nine dogs where bacteria other than *E. coli* were found, all isolates were susceptible to all antimicrobials tested on all sample occasions. In one dog, *S. pseudintermedius* susceptible to penicillin was isolated from the first and penicillin resistant *S. pseudintermedius* from the second sample occasion.

## Discussion

The seven urinary pathogens isolated in this study are the bacterial pathogens most frequently associated with UTI in dogs [21-23]. In accordance with previous reports, *E. coli* was the most frequently cultured pathogen, followed by staphylococci, with *S. pseudintermedius* being more prevalent than *S. aureus* [2,21,24-27].

To avoid false-positive and false-negative results immediate culture after collection of a urine specimen is recommended. If this is not possible, the samples should ideally be refrigerated within 1 or 2 hours of collection [27,28]. In a practice setting urine is however often referred to an external laboratory for confirmation and identification of specific bacterial organisms [27,29]. Furthermore, it is not uncommon for both cystocentesis and second-best options in trying to avoid bacterial contamination of the sample, like catheterization or securing a midstream sample, to be unrealistic alternatives when colleting the sample due to either cost or practical problems in handling of the individual dog. The possible influence on the results of collection method and handling of the samples in the present study could not be fully evaluated as the collection methods were unknown, but prevalence of growth of the individual bacterial species was compared between the two categories pure and contaminated cultures. As previously reported, *E. coli* was found significantly more often in pure culture [30]. This indicates that the relatively high prevalence of *E. coli* in the material was not due to misinterpretation of faecal or genital contamination of samples.

The finding that samples submitted to the laboratory as pre-incubated agar plates or dipslide cultures yielded significantly more positive culture results than non-incubated urinary samples might be explained by attending veterinarians choosing not to submit agar-plates until growth of suspected pathogens is evident. Choice of material was not shown to be a major factor influencing the relative prevalence of bacterial species among the positive cultures, with the exception of staphylococci, which were found more often in pre-incubated sample media. Ling et al. [24] reported that *Proteus* spp. were isolated more frequently from urine specimens collected by catheterization or midstream catch than by cystocentesis, but to what extent the prevalence of various bacterial agents is influenced by sample technique and sample material is otherwise not well described. Further studies specifically designed to compare sample methods and sample materials are warranted.

In the present study, there was a possibility of more than one pathogen causing the UTI in two per cent of all the positive samples. This is in accordance with previous studies showing that more than 70% of UTIs in dogs are caused by a single pathogen [8,24,29]. None of the samples with multiple pathogens originated from the 45 dogs that were sampled repeatedly. This is in contrast to previous studies, where an association between a higher prevalence of multiple organisms and recurrent and persistent UTIs has been reported [21,31].

Antimicrobial susceptibility of *E. coli* from UTIs in dogs in Sweden has previously been reported from samples collected in 1991–92 and in 2002–03 [32]. If our data are re-evaluated by the cut-off values used by Hagman & Greko, the proportions of susceptible isolates in the two studies are similar [32]. Resistance in *E. coli* collected from UTIs in dogs in Sweden is apparently stable or improving, which is supported by national surveillance data showing that susceptibility to relevant antimicrobials has remained stable for the last two decades [33]. The situation is favourable in comparison to studies from other countries, where the proportion of susceptible *E. coli* is smaller [30,34,35].

Susceptibility among *Klebsiella* spp. or *Proteus mirabilis* was high compared to previously reported data [34]. There were several appropriate first line antimicrobials available for treatment in the approximately ten per cent of UTIs caused by either of these two species. The constitutive resistance to nitrofurantoin in both species, to ampicillin and amoxicillin in *Klebsiella* spp. and to tetracycline in *Proteus mirabilis* is reflected in this study as well as in previous reports [34,36].

Among the gram-positive isolates, susceptibility to penicillin and aminopenicillins was uncommon in staphylococci, while all streptococci were susceptible to these antimicrobials. In staphylococci, the second most common cause of UTI, susceptibility to other antimicrobials was high, ranging between 67 and 100%, including 97% of the isolates being susceptible to amoxicillin-clavulanic acid. Our findings agree with previous reports [26,34] and with data regarding the susceptibility of staphylococci and streptococci from dogs in Sweden reported in the monitoring program SWEDRES-SVARM [33]. The inherently low susceptibility of streptococci and enterococci to enrofloxacin that was demonstrated in this study has also been observed by others [34,36]. Several relevant treatment options were thus available when the UTI was caused by a gram-positive pathogen, including the 4% of UTIs caused by enterococci, which was the pathogen with the overall least favourable susceptibility pattern.

Many previous reports on growth of pathogens and antimicrobial susceptibility patterns in urinary tract infections in dogs are based on samples collected at referral animal hospitals based at universities in various countries. The samples included in the present study were from a wide range of veterinary practices with a geographical spread over the country and with both primary care facilities and referral animal hospitals represented, making the material representative of the actual population in the country. Interestingly, the probability of finding less susceptible isolates in our study was significantly higher in samples originating from small animal clinics or mixed veterinary practices than in samples from referral animal hospitals. The only exception to this was amoxicillin-clavulanic resistance in the limited number of *Enterococcus spp*. isolates. It has previously been stated that, as referral hospitals are more likely to have a higher caseload of complicated and recurrent cases, less susceptible isolates should be expected in samples originating from referral animal hospitals compared to smaller clinics [24,25,32].

In accordance with previous studies, *E. coli* was the most common pathogen in the repeated samples [8,21,22]. The national guidelines states that culture and antimicrobial susceptibility testing always is to be performed when UTI is suspected in a dog, the only possible exception being uncomplicated first-time UTIs in young bitches (http://www.smadjurssektionen.se/start/policys-och-guidelines). It is still possible that more dogs had been diagnosed with UTI previously, as other veterinary care facilities in the country do not routinely submit their samples for cultures to SVA. However, the low percentage of recurrent or persistent UTIs correlates with previous reports that concluded that the majority of UTIs in dogs are uncomplicated and occur as single episodes [8,21,22].

Multiresistance in *E. coli* isolates was twice as common in the repeatedly sampled 40 dogs compared to the dogs that were only sampled once. These findings support the need for culture and susceptibility testing and the need for investigating the possible underlying causes in dogs with recurrent or persistent UTI. However, to presume that the cause of a recurrent or persistent UTI is a resistant uropathogen needing combination therapy with broad-spectrum antimicrobials would be incorrect in more than 80% of the cases in the present study.

Phenotypic resistance to ESC in Enterobacteriaceae was rare. A similarly low ESC resistance in *E. coli* from dogs in Sweden was reported during 2010 – 2012 in SVARM, where approximately 1% of *E. coli* from UTIs in dogs had reduced susceptibility to cefotaxime (MIC > 1 mg/L) [33]. Higher prevalence of ESC resistant *E. coli* from various infections sites in dogs has been reported from other countries [4,34,37] and transmissible genes coding ESC resistance known to be common in isolates from humans have been found in bacteria isolated from pet animals [4,38,39]. In the present study, the only transmissible gene demonstrated was *bla*_CMY2_, which in a contemporary study was found in only a small proportion of ESC resistant *E. coli* from humans in Sweden [33]. This scenario might change if ESC resistance becomes common in dogs, and vigilance towards ESC resistance in animal healthcare is warranted.

## Conclusions

*E. coli* was the most frequent pathogen identified in the study followed by staphylococci. *E. coli* was found more often in pure culture than in contaminated samples. Choice of sample material might influence the possibility of detecting *Staphylococcus pseudintermedius* and *Staphylococcus aureus* isolates in clinical cases of UTI in dogs, as S. *pseudintermedius* and *S. aureus* were more prevalent in samples submitted to the laboratory as pre**-**incubated sample media compared to samples submitted as non**-**incubated media.

The results of the susceptibility testing were favourable and use of only first-line antimicrobials is based on the study results a rational empirical antimicrobial therapy for the studied dog population. Disregarding constitutive resistance, susceptibility to the majority of the tested first line antimicrobials was common. Multiresistance was rare, and such isolates were all susceptible to at least one antimicrobial relevant for the species and for the treatment of UTI. Isolates were less susceptible in samples originating from small animal clinics or mixed veterinary practices compared to the samples from referral animal hospitals.

Phenotypic resistance to ESC in Enterobacteriaceae was found in one per cent of all positive cultures. Bacteria with transferable ESC resistance were confirmed in only one dog. The gene demonstrated was *bla*_CMY2_.
